# Plant Growth, Yield, and Fruit Size Improvements in ‘Alicia’ Papaya Multiplied by Grafting

**DOI:** 10.3390/plants12051189

**Published:** 2023-03-06

**Authors:** Irene Salinas, Juan José Hueso, Danilo Força Baroni, Julián Cuevas

**Affiliations:** 1 Department of Agronomy, University of Almería, ceiA3, Ctra. Sacramento s/n, La Cañada de San Urbano, 04120 Almería, Spain; 2 Cajamar Experimental Station ‘Las Palmerillas’, Paraje Las Palmerillas 25, El Ejido, 04710 Almería, Spain; 3 Plant Physiology Laboratory, LMGV, Agricultural Science and Technology Center, State University of North Fluminense, Campos dos Goytacazes 28013-602, RJ, Brazil

**Keywords:** *Carica papaya* L., seedlings, grafting, in vitro, clones

## Abstract

Papaya (*Carica papaya* L.) is one of the few fruit crops still propagated by seeds. However, its trioecious condition and the heterozygosity of the seedlings make urgent the development of reliable vegetative propagation procedures. In this experiment, we compared, in a greenhouse sited in Almería (Southeast Spain), the performance of plantlets of ‘Alicia’ papaya originated by seed, grafting, and micropropagation. Our results show that grafted papayas were more productive than seedlings papayas (7% and 4% for total and commercial yield), while in vitro micropropagated papayas were the least productive (28 and 5% less in total and commercial yield than grafted papayas, respectively). Root density and dry weight were both higher in grafted papayas, while the seasonal production of good quality, well-formed, flowers was also enhanced in grafted papayas. On the contrary, micropropagated ‘Alicia’ plants yielded less and lighter fruit despite these in vitro plants blooming earlier and setting fruit at desirable lower trunk height. Less tall and less thick plants and reduced production of good quality flowers might explain these negative results. In addition, the root system of micropropagated papaya was more superficial, while in grafted papayas, the root system was larger and had more fine roots. Our results suggest that the cost-benefit ratio does not favor the choice of micropropagated plants unless elite genotypes are used. On the contrary, our results encourage more research on grafting, including the search for suitable rootstocks for papaya.

## 1. Introduction

Papaya (*Carica papaya* L.) is a tropical fruit crop of growing interest due to its enormous productivity and rapid entry into production. Agricultural statistics indicate an average yield of 30 t ha^−1^, but high variability exists depending on the country and cultivation system. According to FAOSTAT [1], the total world papaya area is 468,731 hectares, mostly distributed in countries with mild tropical climates, but papaya is also grown in subtropical and temperate climate areas, in the latter only possible under protected cultivation. In Spain, papaya cultivation is restricted to the peninsular Southeast and the Canary Islands. Here, papayas are cultivated under plastic greenhouses and produce 120 t ha^−1^ per year. Unfortunately, papaya lifespan in a greenhouse is limited to around 2 years because its rapid growth makes the plants reach the roof of the greenhouses at that time. Very tall papayas also lead to higher costs in harvest and in flower and fruit thinning operations. 

In nature, papaya is dioecious, producing two different specimens: androic plants (producing only staminate flowers) and gynoecious plants (producing only pistillate flowers). However, the appearance of hermaphrodite plants by mutation has allowed their commercial production by directed crosses [2]. Growers much prefer the cultivation of hermaphrodite plants for their higher productivity and better fruit quality [2,3]. Nonetheless, to be sure that at least one hermaphrodite plant per hole is obtained, a grower must transplant 3–4 seedlings per planting hole in order to increase largely the probability of having at least one hermaphrodite plant. Once the seedlings start flowering, the grower inspects the flower’s sex, selects one hermaphrodite plant, and removes the remaining plants from each planting hole. 

This system of seed multiplication presents many drawbacks. The more obvious are, the higher expenses of buying more plantlets than finally necessary; also, the additional costs of the initial cultivation of the surplus seedlings and the negative effects of the competition among them on plant growth [4]. Plant sex determination with molecular markers allow early determination of the gender of papaya seedlings’ gender at the nursery [5]. However, some drawbacks derived from the sexual origin of the plants persist. For instance, seedlings present a juvenile stage that delays their entry into production, while, on the other hand, the sexual origin of these seedlings leads to an important heterogeneity in the field, with very productive specimens but with others too that, with equal management, lack the yield and fruit quality of neighboring plants [6,7]. Consequently, alternative procedures to multiply papaya by vegetative methods are sought. As developed in most fruit crops centuries ago, the vegetative multiplication of papaya can be achieved by grafting, cuttings, and by in vitro culture propagation [8]. 

Despite the very remarkable interest in developing multiplication by grafting [9], the results are not satisfactory with adult material due to the emission of abundant latex at the grafting point [10]. Grafting using juvenile material is, however, possible and successful [6,9,11], although it lacks the advantages of using adult material such as scion. Cutting has a longer history in papaya, although difficulties exist in obtaining good-quality propagules [8,12]. These problems in traditional vegetative multiplication systems led to the development of in vitro multiplication in papaya. This system guarantees plantlets homogeneity but has a higher production cost [13]. 

The objective of this work is to compare in a greenhouse the growth, rooting, yield, and fruit quality in ‘Alicia’ papaya cultivar obtained by seeds, grafting, and in vitro micropropagation in order to recommend a propagation system for protected cultivation.

## 2. Results and Discussion

### 2.1. Plant Growth Conditions 

The monthly average temperature inside the greenhouse ranged from 14.6 °C in January 2021 to 28.1 °C in July 2020. The average monthly minimum temperatures ranged from 10.3 °C in January and February 2021 to 23.4 °C in July 2020, while the monthly maximum temperatures ranged from 21.6 °C in January 2021 to 35.6 °C in September 2019. On the other hand, the monthly mean relative humidity (RH) fluctuated between 59% recorded in June 2019 and 83% in December 2019, being the mean RH of the minima between 34% in June 2019, and 69% in December 2019, while the mean RH of the maxima ranged between 82% measured in March 2021 and 88% in December 2019.

### 2.2. Plant Growth

**Figure 1:**
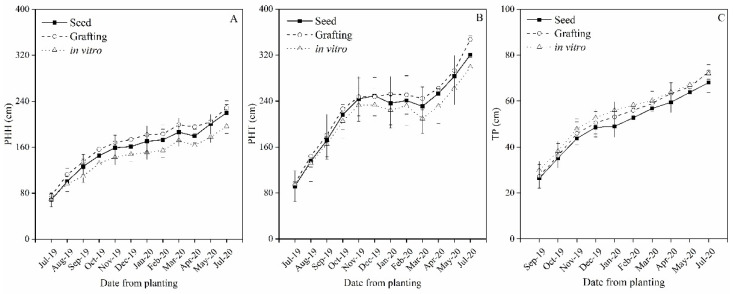
(**A**) Plant height from the ground to the head (PHH); (**B**) Plant height from the ground to the top of the canopy (PHT) and (**C**) Trunk perimeter (TP), in ‘Alicia’ papayas propagated by seed, grafting and in vitro.

‘Alicia’ papayas propagated by seed, grafting, and in vitro followed a similar growth pattern during the first year (Figure 1). However, the in vitro propagated plants grew slower than those propagated by seed and especially those produced by grafting. Although no significant differences were found in the plant height from the ground to the head (PHH), nor in the plant height to the top (PHT; height including erect new leaves), the initial differences against in vitro plants were never reversed (Figure 1A–C). Fitch et al. [4] obtained similar results in Hawaii. This suggests some negative effects for in vitro plants in early establishment and growth. 

All plants grew rapidly during the initial period (June–December 2019), exceeding PHT 200 cm in all treatments (Figure 1B). Reduced growth took place during winter. After this period, the growth was reactivated, reaching the plants with a PHT of 300 cm, in seedlings and in vitro plants and 350 cm in grafted papayas 14 months after planting (Figure 1A,B). On the other hand, the trunks of the plants did not stop growing throughout the study period (Figure 1C). This continuous growth, even in the coldest months of winter, was noticed before [14]. The trunk perimeter reached 70 cm 14 months after planting, less in seedlings (Figure 1C). Fitch et al. [4] compared cuttings, micropropagated, and seedlings plants of ‘Rainbow’ papaya and found no differences neither in plant height nor in trunk growth 9 months after transplanting, suggesting, in their case, good development in all kinds of plantlets regardless the method of propagation. 

**Figure 2:**
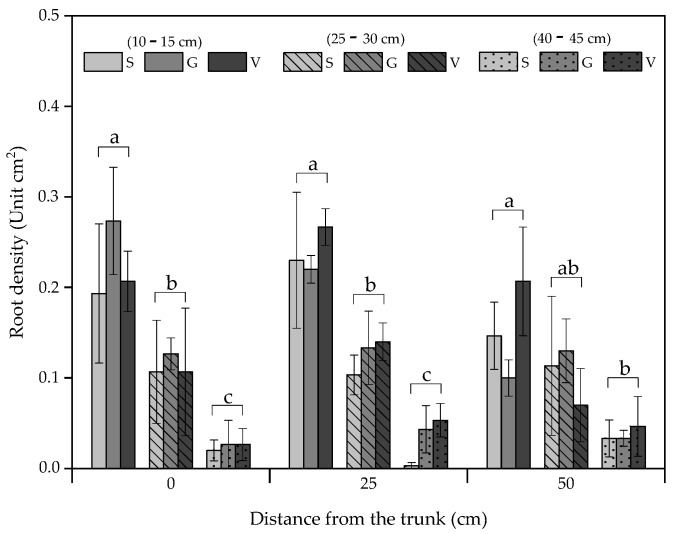
Roots density in ‘Alicia’ papaya propagated by seed (S), grafting (G), and in vitro (V) (average ± s.e.) in different soil depths (10–15; 25–30 and 40–45 cm) and distances from the trunk (0; 25 and 50 cm). Bars followed by different letters indicate statistically significant differences between soil depths at each distance from the trunk.

The root system of the plants obtained by the different propagation procedures was characterized according to root density and dry matter weight. In this regard, no statistical differences were observed between treatments; however, in vitro and grafted plants had higher root density (125 and 121 roots cm^−2^, respectively) than seedlings (106 roots cm^−2^). Heavier root dry matter (3.51 and 3.80 kg per plant) was obtained for the formers compared to 2.80 kg of roots per plant in seedlings. Similar root distribution pattern in depth was observed in all treatments. The highest roots density (205 roots cm^−2^) was found near the surface (10–15 cm), followed by medium depth (25–30 cm) with 114 roots cm^−2^ and finally by the greatest depth analyzed (40–45 cm) (32 roots cm^−2^) with smaller differences among soil depths at the furthest distance from the trunk analyzed (50 cm apart) (Figure 2). Despite no significant differences being found, in vitro papayas showed greater density at 10–15 cm depth (227 roots cm^−2^), evidence of a more horizontal roots distribution (Figure 2), explained by the absence of the radicle, main primary root, developed from seeds. Roots’ dry mass was higher in grafted papayas (Table 1). In this regard, while seedlings developed their roots from hermaphrodite seeds, female seeds were used as a rootstock for grafted papayas. This could explain the small differences in root density and distribution between these two treatments. Papaya grating onto specific rootstocks (selected adult female or male plants) has been used to induce scion vigor and longer lifespan by reducing the risk of soil pathogens infection and by increasing the tolerance to abiotic stresses [5,9].

The lowest insertion points to the trunk for the first flower and first fruit were measured for in vitro papayas, as previously observed by Fitch et al. [4], indicating good fruit sett and less abortion in the first reproductive nodes of the trunk. However, no statistical differences were found among propagation procedures (Table 2). Vegetatively propagated papayas form the first flowers and fruits at a lower height on the trunk than seedlings, especially when several seedlings are initially planted together in a hole [4,6,15]. This behavior is explained by the juvenility of the seedlings. The competition for sunlight between seedlings of the same hole might also play a role since it leads to an increase in the length of the internodes of the stem of seedlings before blooming. 

**Figure 3:**
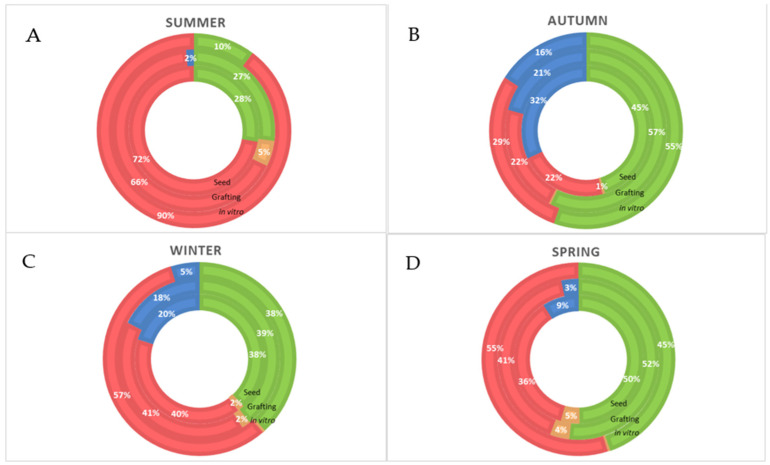
Seasonal frequency (%) of elongata hermaphrodite flowers (green); that later will produce fruits of commercial interest, non-elongate hermaphrodite flowers (orange), pentandric and carpeloid hermaphrodite flowers (red), and sterile; functionally male flowers (blue) found in ‘Alicia’ papaya propagated by seed, grafting and in vitro, and determined in (**A**) summer, (**B**) autumn, (**C**) winter and (**D**) spring, from August 2019 to June 2020.

The seasonal frequency of different floral types was evaluated by inspecting all open flowers on each sampling date. The highest percentage of elongata flowers, hermaphrodite flowers that later will produce commercial fruits, were obtained in all treatments in autumn and spring (Figure 3). Spring and autumn conditions are especially suitable for papayas cultivated in the greenhouses of Almería [7,16]. In contrast, ‘Alicia’ seems poorly adapted to our hot summers, forming more than 70% of misshapen flowers regardless of the propagation procedure used (Figure 3A). Functionally male flowers appeared more in autumn and winter, as well as in spring for grafted and, especially, seedlings papayas (Figure 3B–D). Grafted papayas formed a higher percentage of elongata, good-quality flowers during winter, spring, and autumn (Figure 3). Our results are in line with those obtained in Brazil [17]. In the Brazilian work [17], the formation of carpel loid (cat-face) fruits was related to high temperatures, large thermal amplitude, and exuberant vegetative growth (as happens in summer in our conditions). The higher occurrence of functionally male flowers was also related to poor sunlight and low vigor (as in autumn and winter in our conditions).

### 2.3. Yield

**Figure 4:**
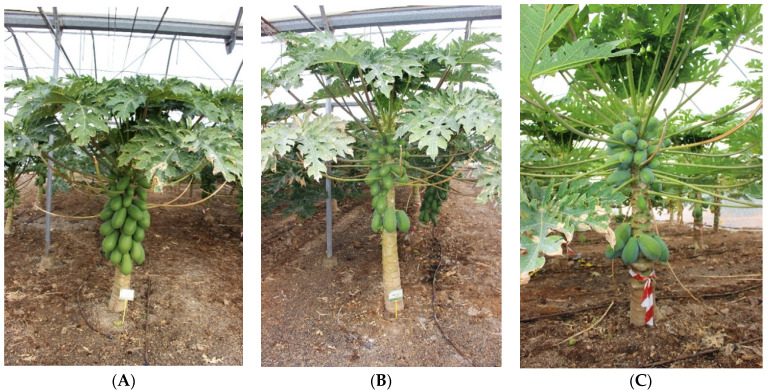
‘Alicia’ papaya plants propagated by (**A**) seed, (**B**) grafting, and (**C**) in vitro.

Harvest started in February or March 2020, depending on the treatment; it was carried out on 10 and 24 February for in vitro and seedlings plants, respectively, and later, on 10 March, in grafted papayas, likely an expression of the higher vigor of the latter. The total and commercial yield was higher (28 and 25% more, respectively) in grafted than in vitro plants. The plants that originated directly from seeds showed intermediate values (Table 3). Discards were low in all treatments since misshapen flowers and fruits were thinned early. Although there was no statistical significance, the differences in yield were due to grafted papayas producing more and heavier fruits than in vitro propagated plants. Araya-Valverde et al. [3] have also observed lower fruit weight in micropropagated papayas. Our results suggest that the less-developed root system in micropropagated plants reduced plant growth (trunk and height), which in turn affected flower quality and fruit growth. That, despite in vitro, plants started producing earlier at a lower trunk height (Figure 4).

Vegetative propagation provides clear advantages, such as higher yield, if selected elite plant material is selected [5,6,18,19]. However, in our study, only grafted papayas were more productive than seed-propagated papayas. Yield increase and better fruit quality were also obtained for the ‘Intenzza’ cultivar when grafted on female seedlings [20], thus confirming the interest in using commonly discarded female seedlings as rootstocks. Lima de et al. [21] compared ‘Golden’, ‘Sunrise Solo’, and ‘Tainung 01’ papayas propagated by grafting and by seed and recommended grafting as the trees were more productive. Grafted trees were shorter, larger in trunk diameter, and had more leaves than seedlings. Fitch et al. [4] suggest that the use of cloned papayas improves yield in areas with lower solar radiation and poor soils but that seed-propagated plants might produce more than vegetatively propagated plants in areas with optimum temperatures and solar radiation and fertile soils. In our conditions, female plants as rootstock in grafted papayas improved plant development as the increase in root dry weight and better root distribution suggest. 

### 2.4. Fruit Quality

In the evaluation of fruit quality carried out in June, fruits from in vitro plants were again smaller and significantly lighter than those harvested from seedlings but significantly sweeter (an average > 9 °Brix) than those from the other two propagation treatments (Table 4). No significant differences were obtained in TA. On the other hand, seedlings produced in June the heaviest fruits (>1.4 kg), with a larger size but also with a too-big cavity (Table 4). Finally, no significant differences were found in fruit firmness nor in skin and pulp color (Table 4).

**Figure 5:**
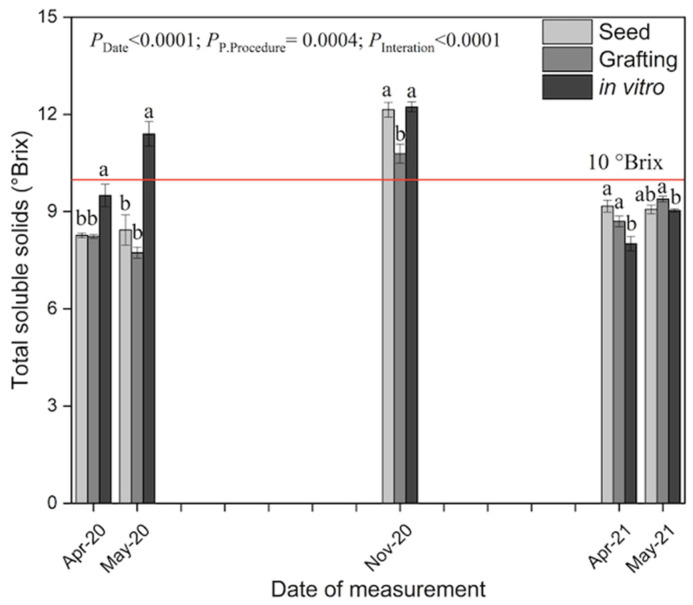
Total soluble solids (TSS) in ‘Alicia’ papaya propagated by seed, grafting, and in vitro (*n* = 12 ± s.e.), evaluated from April 2020 to May 2021 at the maturation of 100% yellow skin color. The line marks the 10 °Brix value, commonly referred to as the minimum for papaya commercialization. Different letters on the same date indicate statistically significant differences between propagation procedures (Tukey’s test *p* < 0.05).

In addition to the fruit quality evaluation in June, the total soluble solids content (TSS) of fruits of the propagation procedures was compared on different dates. In these measurements, we observed that the TSS values were again higher for in vitro plants in April and May of 2020 but lower one year later, although at that time, the differences were slight (Figure 5). In early spring (April), all plants produced papayas with TSS values lower than 10 °Brix at harvest, the level referred to as the minimum required for commercialization in Europe. In May 2020, only in vitro propagated plants produced fruit with a TSS content higher than 10 °Brix. Interestingly, in November, all procedures showed higher TSS. Pinillos et al. [22] found that the harvest period strongly affects the fruit quality of papaya cultivated in a greenhouse. In general, the fruits tend to be lighter and with higher TSS content in autumn than in spring. Salinas et al. [23] obtained the same response working with ‘Intenzza.’

## 3. Materials and Methods

### 3.1. Site and Plant Material

This study was carried out at the Cajamar Experimental Station ‘Las Palmerillas’, located in El Ejido (Almería, Spain) (2°43′ W, 36°48′ N and 151 m above sea level). The papaya crop grew in a multi-tunnel type greenhouse made of galvanized steel, with a 200 μm thick asymmetrical low-density polyethylene cover. The greenhouse consists of eight spans E-W oriented, 7.5 m wide each, with 3.4 m height to the eaves and 5.4 m to the ridge. The trial took place in a southern-oriented individual module composed of four spans (900 m^2^ area).

Growth, yield, and fruit quality of hermaphrodite plants of ‘Alicia’ papaya (CapGen Seeds, Spain), obtained by different propagation procedures, were compared. Seed-propagated plants, selected in a sexing process based on the use of molecular markers, were compared to plants obtained by vegetative propagation procedures, specifically grafting and in vitro multiplication (Figure 4). For grafting, we used hermaphrodite plants of ‘Alicia’ as scions grafted onto rootstocks constituted by female plants of the same cultivar [8]. The third treatment was constituted by plants produced by in vitro culture provided by Vitalplant, S.L. nursery (Spain). The plantation spaced 2.5 × 2.5 m, was carried out with small plantlets of equal size on May 2019, and was terminated in October 2021. Orchard management was the same for all plants regardless of propagation procedure. In this respect, irrigation and fertilization were equally performed in all treatments. Misshapen flowers and non-commercial fruits were removed early, as were the blades of senescent leaves. Pests and diseases were controlled following Integrated Pest Management guidelines.

### 3.2. Plant Growth Conditions

Natural ventilation through a zenithal window per span and a lateral panel improved climate conditions inside the greenhouse. The temperature to open greenhouse windows was managed through a Priva weather controller and set at 24 °C. In addition, roof whitening was performed to improve the climate conditions inside the greenhouse in summer. The greenhouse climate was regulated with a cooling system consisting of a low-pressure nebulization system CoolNet Pro (Netafim), with 5.5 L h^−1^ double emitter spaced 0.1 nozzles m^−2^ and 1 L m^−2^ h^−1^ flow, activated when the relative humidity was below 60%. A heating system consisting of a hot flow air Ermaf RGA95 (Elster), with diesel as fuel, was activated during cold periods (from November to March) when the temperature was ≤12 °C. Temperatures and relative humidity inside the greenhouse were recorded during the experiment.

### 3.3. Plant and Fruit Measurements

**Figure 6:**
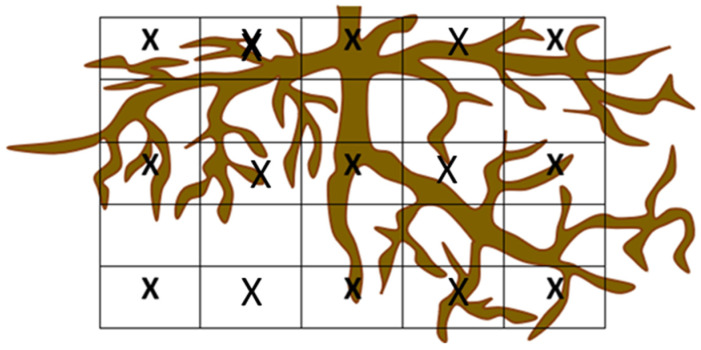
Representation of the mesh used to measure root density at different soil depths and distances from the trunk. Each quadrant measures 20 cm^2^. ‘X’ represents the 15 sampling points per plant.

**Figure 7:**
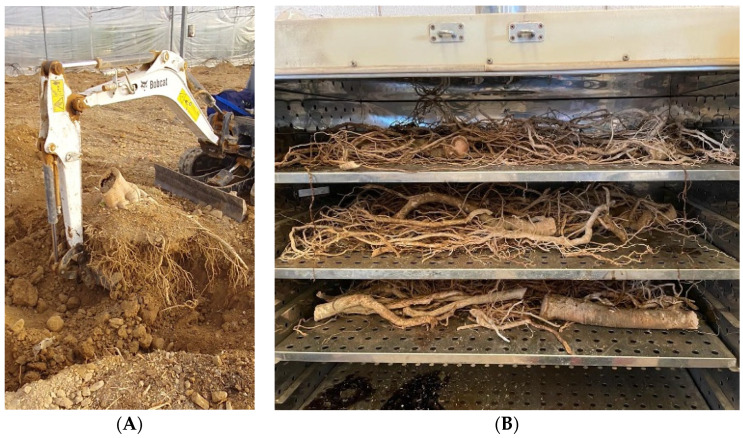
(**A**) Root extraction and (**B**) drying at 65 °C of ‘Alicia’ papaya plants propagated by seed, grafting, and in vitro.

Plant height from the ground to the head (PHH) and from the ground to the top of the canopy (PHT), that is, including erect leaves, were measured monthly using a graded bar. The trunk perimeter (TP) at 15 cm from the ground was measured with the same periodicity using a tailor tape ruler. After pulling up the plants in November 2021, the power and extension of their root system were studied. The root analyses proceeded as follows: a trench (40 cm wide × 100 cm long) was made in the east and west positions of the plants, 50 cm away from the trunk. Then, the roots in the trench were counted with the help of a mesh with 10 cm^2^ subareas at three different soil depths (10–15; 25–30, and 40–45 cm) and later similarly at three distances from the trunk (0, 25 and 50 cm), obtaining thus the root density at each soil depth and trunk distances on both cardinal sides (East and West) (Figure 6). Then, the average between both cardinal sides was calculated for each soil depth and trunk distance. Finally, the soil was excavated using a mini-tractor (Figure 7A) at one meter around each plant to extract the maximum number of roots. The extracted roots were washed and placed in an oven at 65 °C for 120 h (Figure 7B) to estimate the total dry mass of the roots expressed as kilograms per plant.

**Figure 8:**
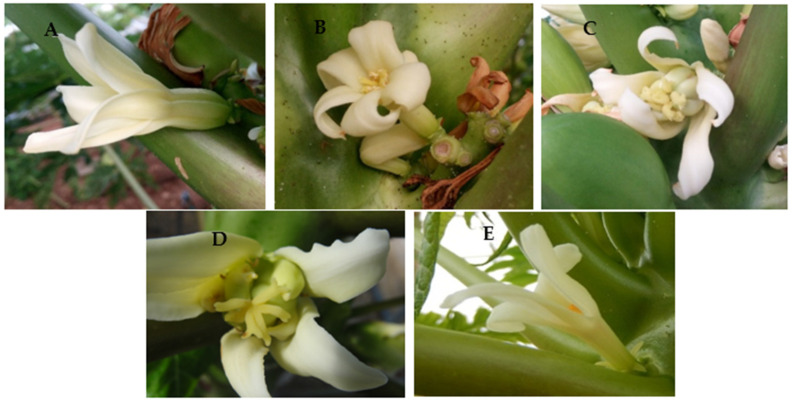
Main flower types described in papaya, evaluated in ‘Alicia’ papaya propagated by seed, grafting, and in vitro: (**A**) elongata flowers (those that will produce commercial fruits); (**B**) non-elongata, (**C**) pentandric, and (**D**) carpeloid misshapen flowers; and (**E**) functionally male flowers.

The distance from the ground to the first flower and to the first fruit was measured using a tailor tape ruler. All flowers in anthesis were inspected from the beginning to the end of the blooming period (August 2019 to June 2020) to determine the frequency of elongata flowers (those that produce fruits with commercial value) and also of non-elongata, pentandric, and carpeloid misshapen flowers, and of functionally male flowers (Figure 8). The seasonal average of each type of flower was calculated from these data. Harvest was performed when fruits reached 30–50% yellow color in the skin. Total and commercial yield, discards (fruits lighter than 200 g and non-commercial fruits), the number of fruits, and their average fruit weight were compared among treatments.

Fruit quality was evaluated on 5 June 2020, just before the end of the trial. For this, one fruit per plant (that is, 12 fruits per treatment) was collected at 50% of yellow skin and analyzed four days later, when they showed their skin 100% yellow. Fruits were weighed on a balance (d = 0.1 g) (model SB12001, Mettler Toledo, Barcelona, Spain), and their longitudinal and equatorial perimeter were measured using a tailor tape ruler, as it was the cavity width, with a digital caliper (model Z22855, PowerfixProfi, Neckarsulm, Germany). Pulp firmness was determined on each fruit in two opposite equatorial points using a digital penetrometer (model DFT 14, Agro Technologie, Forges Les Eaux, France) after removing the skin of the fruit. Skin and pulp color were determined in three positions of each sampled fruit, with the help of a colorimeter (model CR-400, Konica Minolta, Co., Tokyo, Japan). These results were expressed considering hue angle (hue°), which indicates the color tone of the fruit so that a hue angle of 0° corresponds to red color, 45° orange color, 60° to yellow-orange color, 90° to yellow color and 120° to yellowish green color. Total soluble solids content (TSS) and titratable acidity (TA) of the juice of the fruits were also evaluated. TSS was measured using a digital refractometer (model PR-101, Atago Co., Tokyo, Japan), while TA was assessed by titration with 0.1N NaOH, using phenolphthalein as an indicator, and expressed as grams of citric acid per liter of juice. In addition, TSS has compared during the experiment. 

### 3.4. Statistical Assessment

A randomized complete block with three treatments (seedlings, grafted, and in vitro plants) and three replicates was designed. A tree row per treatment constituted each replicate, in which the four central trees per row (12 plants per treatment) were selected for measurements, thus avoiding border plants. The data were subjected to analysis of variance (ANOVA), and the means were separated by Tukey’s Test using R software version 4.2.1 (R Core Team 2020, Vienna, Austria).

## 4. Conclusions

Our results show that micropropagated ‘Alicia’ plants produced less and lighter fruit, despite these plants bloomed earlier and set fruit into a lower trunk height. Less tall and less thick plants with a reduced production of good-quality hermaphrodite flowers might explain the negative results. More superficial root distribution in micropropagated plants has penalized too this treatment in terms of total and commercial yield. On the contrary, grafted papayas performed better than micropropagated plants and than seedlings, without differences with the latter above ground, because no selected (elite) material was used as scion. The difference between them is that in seedlings, the root system is from hermaphrodite plants, while in grafted plants, the root system comes from female seedlings, and the latter had more root density and a larger root system. 

Further confirmation of our results might open a new vegetative procedure for producing more productive papayas. Knowing that obtaining propagules as rootstocks from mother plants is costly and that rooting plants in vitro are not easy nor cheap, our results encourage more research on grafting, including the search for suitable compatible rootstocks for papaya. We initiate this search within the genus *Vasconcellea* and the species *Carica pentagona*.

## Figures and Tables

**Table 1:** Roots dry mass (average ± s.e.) in ‘Alicia’ papayas propagated by seed, grafting, and in vitro at the end of the experiment.

Propagation Procedure	Roots Dry Mass (kg plant^−1^)
Seed	2.80 (±0.95) ^ns^
Grafting	3.80 (±0.73)
in vitro	3.51 (±0.15)
*p* value	0.612

^ns^ No statistical differences at *p* less than 0.05.

**Table 2:** Distance from the ground to the first flower and to the first fruit (average ± s.e.) in ‘Alicia’ papaya propagated by seed, grafting, and in vitro.

Parameter	Propagation Procedure		
	Seed	Grafting	In Vitro
Distance to the first flower (cm)	62.6 ± 5.70	59.9 ± 5.78	56.7 ± 2.73
Distance to the first fruit (cm)	87.9 ± 6.90	91.7 ± 2.57	75.8 ± 7.91

Without significant differences in any parameter among propagation procedures.

**Table 3:** Total and commercial yield, discards, fruits number, and weight (average ± s.e.) in ‘Alicia’ papaya propagated by seed, grafting, and in vitro.

Parameter	Propagation Procedure		
	Seed	Grafting	In Vitro
Total yield (kg m^−2^)	16.9 ± 2.18 a	18.1 ± 2.09 a	14.1 ± 1.76 a
Commercial yield (kg m^−2^)	15.9 ± 2.25 a	16.6 ± 2.00 a	13.3 ± 1.57 a
Discards (%)	5.9 ± 1.13 a	8.3 ± 1.21 a	5.7 ± 0.72 a
Fruits per m^2^	12.5 ± 1.29 a	12.8 ± 1.63 a	11.2 ± 0.98 a
Fruit weight (g)	1250 ± 66.94 a	1310 ± 12.58 a	1174 ± 43.82 a

Different letters in the same row indicate statistically significant differences between propagation procedures (Tukey’s test *p* < 0.05).

**Table 4:** Fruit size and quality at maturation (100% yellow skin color) (average ± s.e.) in ‘Alicia’ papaya propagated by seed, grafting, and in vitro, evaluated on 5 June 2020 on 12 fruits per treatment.

Parameter	Propagation Procedure		
	Seed	Grafting	In Vitro
Weight (g)	1435 ± 59.64 a	1285 ± 62.45 ab	1188 ± 56.58 b
Longitudinal perimeter (cm)	53.6 ± 0.84 a	51.8 ± 0.87 a	51.1 ± 0.86 a
Equatorial perimeter (cm)	38.4 ± 0.52 a	37.1 ± 0.63 ab	35.4 ± 0.55 b
Cavity width (cm)	5.9 ± 0.14 a	5.5 ± 0.23 ab	4.9 ± 0.13 b
Firmness (N)	27.6 ± 4.71 a	13.0 ± 2.64 a	27.2 ± 7.77 a
TSS (°Brix)	7.2 ± 0.27 b	7.6 ± 0.17 b	9.1 ± 0.28 a
TA (g citric acid L^−1^)	1.0 ± 0.06 a	0.9 ± 0.05 a	0.9 ± 0.06 a
Skin color (hue°)	90.0 ± 4.72 a	83.4 ± 1.55 a	86.0 ± 1.23 a
Pulp color (hue°)	60.5 ± 0.85 a	60.6 ± 0.66 a	61.9 ± 0.39 a

Different letters in the same row indicate statistically significant differences between propagation procedures (Tukey’s test *p* < 0.05). TSS: Total soluble solids; TA: Titratable acidity.

## Data Availability

Data are available on requests to authors.
